# Comparative Electromyographic Activity of Hamstrings During Sprinting Versus Strengthening Exercises: Implications for Injury Prevention

**DOI:** 10.7759/cureus.66370

**Published:** 2024-08-07

**Authors:** Ayrton Moiroux-Sahraoui, Jean Mazeas, Emine-Anika Bener, Ismail Bouzekraoui Alaoui, Maurice Douryang, Florian Forelli

**Affiliations:** 1 Sports Rehabilitation, Orthosport Center, Domont, FRA; 2 Orthopaedic Surgery, Clinic of Domont, Domont, FRA; 3 Research Unit, Mohammed VI Center for Research and Innovation, Rabat, MAR; 4 Mohammed VI Faculty of Nursing and Allied Health Professions, Mohammed VI University of Health Sciences, Casablanca, MAR; 5 Department of Physiotherapy and Physical Medicine, University of Dschang, Dschang, CMR; 6 Research Unit, Société Française des Masseurs Kinésithérapeutes du Sport (SFMKS) Lab, Pierrefitte-sur-Seine, FRA

**Keywords:** prevention, emg, isokinetics, nordic hamstring, sprint, injuries, hamstrings

## Abstract

Background: Hamstring injuries are a major problem in sports involving sprinting, such as soccer, rugby, and track and field, and lead to sports stoppages and psychological, social, and financial repercussions. For several years now, these injuries have been stagnating or even increasing. Preventing these injuries is therefore a fundamental issue for at-risk athletes. The aim of this study was to compare the electromyographic (EMG) activity of the hamstrings in athletes during sprinting, Nordic hamstring exercise (NHE), and high-speed concentric exercise on an isokinetic dynamometer.

Methods: A pilot study was conducted on a population of 15 sprint-exposed field athletes (22.54 ± 3.71 years, Tegner score ≥ 6) with no history of hamstring injury in the last six months. The protocol included a warm-up, followed by three repetitions of the NHE, two sets of 10 repetitions on the isokinetic dynamometer at 300°/sec, and a maximal sprint. Exercises were randomized for each subject, and muscle activity was assessed using wireless EMG sensors during each test. EMG data were normalized to the measured maximum voluntary isometric contraction (MVIC), and test results were statistically analyzed to determine which exercise resulted in maximal hamstring activation.

Results: Comparison of hamstring muscle activity between exercises showed a significant difference for most of our results (p-value < 0.05). The results show significantly higher mean hamstring activity during sprinting (0.4800 ± 0.19 μV) compared with strengthening exercises. The NHE (0.3201 ± 0.09 μV) was the second most active exercise. In the last place was the high-speed concentric exercise on the isokinetic machine, which produced less activation than the other exercises (0.2487 ± 0.07 μV).

Conclusions: Sports involving sprinting are at risk of a hamstring injury but it appears that its use in rehabilitation and prevention of hamstring injury is relevant, as it would allow high-intensity muscle activation to prepare the hamstring for this type of loading. However, it is also fundamental to integrate strengthening exercises such as NHE in combination with sprinting in our rehabilitation. Finally, the use of the isokinetic dynamometer does not constitute a first-line choice for hamstring injury management.

## Introduction

Acute myo-collagenous hamstring injuries are among the most common non-contact injuries with short- and long-term consequences in athletes [1]. Their incidence has been rising steadily in recent years, with an average annual increase of 10 to 20% between 2001 and 2022 in European soccer teams [2].

High-speed hamstring injury occurs particularly when the athlete reaches a maximum speed of between 90 and 100% or during acceleration. In most cases, they involve the long head of the biceps femoris [3].

Electromyographic (EMG) analysis during sprinting confirms the importance of the hamstrings. Indeed, there is significant activation of the hamstrings during the propulsion phase (late stance phase), but it is at the end of the oscillation phase that we find the peak of EMG activation (late swing phase) [4]. Coupled with this EMG activation peak, we also note that the end of the swing phase corresponds to the moment when the hamstrings are most stretched [5]. They must, therefore, produce significant force at a time when they are very stretched. By combining these two phenomena, it is easier to understand why this muscle is subject to so many injuries. Current work is also very poor on the activation patterns of the hamstrings during commonly used strength training exercises [6].

There are differences in activation during running in athletes with a history of hamstring injury, particularly in the terminal phase of sprinting. At the same time, during the "late swing phase," a higher knee flexion angle and a decrease in the long head of the biceps femoris muscle length were noted in the injured limb [7].

The most used eccentric exercise for hamstring injury prevention is the Nordic hamstring exercise (NHE). NHE has been shown to induce greater activation of the hamstrings compared with a selection of exercises commonly used in resistance training and injury rehabilitation [8].

The isokinetic dynamometer can be useful as a muscle-strengthening tool. It enables precise work to be carried out in terms of amplitude, intensity, and speed [9-11], enabling the development of rehabilitation programs for knee injuries and imbalances [12].

The primary objective of this study was to analyze and compare the peak and average EMG activity of the hamstrings during sprinting, NHE, and high-speed concentric exercise on an isokinetic dynamometer to determine which exercise induces the highest hamstring activation levels in athletes. The secondary objective is to provide rehabilitation specialists with detailed EMG activity data for the hamstrings to guide the selection of effective exercises for hamstring injury prevention and rehabilitation programs, emphasizing exercises that generate the highest muscle activation.

## Materials and methods

Study design

For this pilot study, a file was filed with the National Commission for Information Technology and Civil Liberties (CNIL), bearing the number 2224821. Subjects participating in the study were informed of the protocol's progress through an explanatory document acting as informed consent after signature. Data obtained during the study were anonymous. The examinations carried out were free, non-invasive, and non-hazardous for the subjects.

Sample size calculation

If we estimate the target population at 8,319 people [13], i.e., the number of athletes with a hamstring injury over one year, tolerating a margin of error of 5% and assuming a confidence level of 95%, the sample size required to obtain potentially significant results is 381 participants. If the study includes 15 participants, within feasible limits, the margin of error will be 25%.

Participants

We evaluated 15 subjects with no hamstring history in the last six months and who were able to perform sprints. Patients were included if they had no history of trauma in the last one year, had no hamstring injury for more than six months, including recurrence, were able to sprint, did at least eight hours of weekly training, including repetitive sprinting (soccer, rugby, athletics), and had an activity level greater than or equal to 6 on the Tegner scale [14].

The general exclusion criteria were subjects who had suffered a hamstring injury in the previous six months and those with pain or discomfort sufficient to influence their ability to sprint or perform any of the study exercises. Also excluded from the study were subjects with lower limb pain greater than 2 on the visual analog scale. In addition, subjects taking part in the study were instructed not to take any psychotropic substances prior to the various tests.

The subjects represent a population of athletes aged 18 years or over who can perform a pain-free sprint.

Eligible patients’ baseline information, demographic data (age, body mass, sex, dominant lower limb), and sports participation data (Tegner scores) were recorded.

Randomization and blinding

Subjects participating in the study were not informed of the study's hypotheses and data. No feedback on values or protocol was given to patients. However, it is impossible to rule out the possibility that they had no contact with each other outside the sessions in which the data were measured. Physiotherapists, for their part, have not been blindsided. The order of study exercises was randomized for each subject using the Pickme application, but groups were not randomized.

Muscle maximal isometric torque

The patients were seated for the evaluation of their quadriceps maximum voluntary isometric contraction (MVIC). Their MVIC was assessed with the knee flexed at 45°. Manual resistance was added by the physiotherapist above the malleoli to avoid interfering with the ankle joint. Subjects were instructed to "bring their heel down as far as possible to the level of their buttocks." Subjects performed three maximal isometric contractions of the hamstring in the prone position for six seconds, with 20 seconds of rest between each repetition. During testing, participants were verbally encouraged to push as hard as possible. The highest torque achieved over the three tests was used as the MVIC and the corresponding highest EMG signal was also used for further analysis.

Outcome measure - surface electromyography

The activation of the hamstring was measured by surface EMG during the MVIC testing. Electrodes were positioned according to the recommendations of SENIAM (Surface EMG for Non-Invasive Assessment of Muscles) [15] to avoid overlap and cross-talk of the innervation zones and muscles, respectively. The skin of the thigh was shaved, abraded, and cleared with alcohol. Active bipolar electrodes, with 20 mm between the poles, were placed longitudinally over the semitendinosus, and biceps femoris muscle bellies to record the electrical activity of the hamstring. A line was drawn between the ischial tuberosity and the medial epicondyle of the tibia, and the first EMG was placed in the middle of this line. A second line was drawn between the ischial tuberosity and the lateral epicondyle of the tibia, and the second EMG was placed in the middle of this line. The patients were laid prone with the knee flexed at 90° for EMG recording.

FREEEMG® surface electromyography (BTS Bioengineering, Garbagnate Milanese, Italy) is an experimental assessment tool that enables the recording and analysis of myoelectric signals. It is one of the non-invasive methods for recording neuromuscular activity. It is said to be surface-based because the pre-gelled electrodes that collect the signal are placed directly on the skin opposite the muscle to be studied. Myoelectrical signals are produced by physiological variations in the membrane of muscle fibers during nerve stimulation of muscles during contraction. We used the BTS EMG-Analyzer 2.9.40.0® software (BTS Bioengineering), which enabled us to process the information gathered and display the results in graphical form. The software was installed on an ASUS 15.6" M570DD-DM061T (Taipei, Taiwan) laptop (Nvidia GeForce GTX 1050 graphics, Nvidia Corporation, Santa Clara, CA) [16].

Intervention

Initially, each subject warmed up with a five-minute run on the NordicTrack® ELITE 5000 treadmill (Logan, UT) at a speed between 8 and 10 kph. Subjects were then asked to perform athletic routines such as knee raises, heel and buttock raises, and straight leg raises. They were asked to perform two roundtrips for each exercise over a 15-meter distance marked out by studs. Finally, they were asked to perform three acceleration phases over 10 meters. Subsequently, the EMG electrodes were positioned to measure the muscle activity and remained attached throughout the intervention. Electrodes were placed after the warm-up. They were placed according to the SENIAM guidelines on the dominant limb of each subject. Following the MVIC measurement, participants performed, in a random order previously defined by the Pick Me® application (version 2022.2), a Nordic hamstring test, a high-speed isokinetic test, and a 30-meter sprint. Rest periods were set up between each series.

The sprinting exercise involved three maximal 30-meter sprints with a five-minute rest between each sprint. The NHE was performed by having participants kneel on a padded surface with their ankles secured and gradually lowering their torso forward. Each participant performed three sets of five repetitions with a two-minute rest between sets. For the high-speed concentric exercise on an isokinetic dynamometer, participants performed three sets of 10 maximal knee flexions at a speed of 300 degrees per second, with a two-minute rest between sets.

Statistical analysis

Statistical analyses were carried out using Excel® spreadsheets (Microsoft Corporation, Redmond, WA) and RStudio® (2022.02.1+461) software (Posit, Boston, MA). The level of significance was set at 0.05.

First, we performed descriptive statistics with the mean and standard deviation on the age, height, weight, and gender of our test group. Secondly, a descriptive analysis was performed with the mean and standard deviation for electrical activation of the biceps femoris and semitendinosus chiefs during each exercise. These data were used to perform statistical tests. Due to the small sample size, a parametric test, the Student's t-test, was used to obtain the statistical results of our data.

For each item, the distribution of most of our tests followed a normal distribution. We used the Student’s t-test. We then set out to compare exercise activities in pairs. We carried out tests on three comparisons: high-speed isokinetic exercise compared with NHE, NHE compared with sprint, and high-speed isokinetic exercise compared with sprint.

## Results

Demographic and baseline characteristics

The mean age of the participants was 22.54 ± 3.71 years. A summary of baseline data (age, BMI, gender, and dominant lower limb) is presented in Table 1.

**Table 1:** Demographic and baseline characteristics. Note: All values are presented as mean ± standard deviation, unless stated otherwise. y: years; m: meters; kg: kilograms; M: male; R: right.

	Test group (n = 15)
Age (y)	22.54 ± 3.71
Body mass index (kg/m^2^)	13.85 ± 8.28
Sex (M)	13
Dominant (R)	11
Tegner score	6.2 ± 1.1

Comparison between high-speed isokinetic exercise and NHE

A non-significant difference (p-value > 0.05) for the biceps femoris (BF) chief was noted, although we did notice a certain tendency toward increased activity. However, we can read a statistically significant difference (p-value < 0.05) for the semitendinosus (ST) chief and the averages of the two muscle chiefs (Table 2).

**Table 2:** Comparative table of EMG activity and high-speed isokinetic testing and NHE. p: two-tailed Student's t-test; significance threshold for p-value: p < 0.05. Values are expressed as mean ± standard deviation (SD). EMG: electromyographic; NS: non-significant difference; Iso: isokinetic test; BF: biceps femoris; ST: semitendinosus; NHE: Nordic hamstring exercise.

	Iso	NHE	p-value
BF	0.24 ± 0.08	0.27 ± 0.09	NS
ST	0.26 ± 0.1	0.37 ± 0.14	0.03
mean	0.25 ± 0.07	0.32 ± 0.09	0.02

Comparison of NHE and sprint exercises

No significant difference (p-value > 0.05) for the ST head was noted, except in the one-sided Wilcoxon test. We can read a statistically significant difference (p-value < 0.05) for the BF chief and the averages of the two muscle chiefs (Table 3).

**Table 3:** Comparative table of NHE and sprint EMG activity. p: two-tailed Student's t-test; significance threshold for p-value: p < 0.05. Values are expressed as mean ± standard deviation (SD). EMG: electromyographic; NS: non-significant difference; BF: biceps femoris; ST: semitendinosus; NHE: Nordic hamstring exercise.

	Sprint	NHE	p-value
BF	0.48 ± 0.20	0.27 ± 0.09	<0.01
ST	0.48 ± 0.21	0.37 ± 0.14	NS
Mean	0.48 ± 0.19	0.32 ± 0.09	0.02

Comparing high-speed ISO with sprinting

A statistically significant difference (p-value < 0.05) for each of the muscle chiefs, as well as for the average of the two chiefs was noted (Table 4).

**Table 4:** Comparative table of EMG activity in isokinetic testing and sprinting. p: two-tailed Student's t-test; significance threshold for p-value: p < 0.05. Values are expressed as mean ± standard deviation (SD). EMG: electromyographic; Iso: Isokinetic test; BF: biceps femoris; ST: semitendinosus.

	Sprint	Iso	p-value
BF	0.48 ± 0.20	0.24 ± 0.08	<0.01
ST	0.48 ± 0.21	0.26 ± 0.1	<0.01
Mean	0.48 ± 0.19	0.25 ± 0.07	<0.01

## Discussion

This study demonstrated that EMG activation of the hamstrings during sprinting is greater than during NHE and high-speed isokinetic exercise. Moreover, hamstring electrical activity is greater during NHE than during high-speed isokinetic exercise.

The results of our study allow us to conclude that NHE enables significant activation of the hamstrings, although this does not match the ability of sprinting to record maximum EMG activation. The study by Prince et al. concurs with our findings regarding EMG activation during NHE and sprinting. This study shows that none of the exercises tested induced hamstring EMG activity greater than 60% of the maximum activity measured during sprinting. The authors suggest that high-speed running and sprint work are essential during prevention and rehabilitation. Despite a population difference between our studies and an absence of isokinetic exercise, these results are in line with our study, where the average activation of the sprint was higher than our other two exercises [17].

In a 2012 study, Iga et al. [8] analyzed EMG activation of the hamstrings during NHE and changes in the contractile function of this muscle group after training for this exercise. The authors observe that the dominant and non-dominant limbs of participants are similarly engaged during NHE and that EMG activity of the ST and BF is elevated during exercise. They also stipulate that NHE training resulted in gains in hamstring performance in both limbs in these footballers. The authors therefore suggest that, although NHE is performed at low speeds, the training results in adaptations that are transferable to high-speed movements such as sprinting. A few authors, such as Freeman et al. in 2019 [18], have looked at the effects of sprint and NHE training on eccentric hamstring strength and sprint performance. The results of this study suggest that sprint training appears to produce sufficient stimulus to improve eccentric hamstring strength. Although NHE produced a significant gain, this improvement was not significantly greater than that of the sprint group. Furthermore, NHE produced no change in sprinting performance, which runs counter to the previously reported study by Iga et al., which observed that NHE produced adaptations that are transferable to high-speed movements [8].

Following on from this, Ditroilo et al. [19] also published a study in 2013 looking at EMG analysis of the hamstrings during two eccentric exercises: the NHE and on an isokinetic dynamometer (30°/s). The results of the study show that the BF is significantly more activated during NHE than during eccentric exercise on an isokinetic dynamometer. It should be noted that the level of activation recorded during NHE varied by two to three times the level of activation during exercise on the isokinetic. NHE led to a mean activation of 0.312 ± 0.120 μV, while eccentric isokinetic exercise produced a mean activation of 0.276 ± 0.146 μV. In addition, they found that eccentric exercise on the isokinetic machine resulted in less activation of the hamstrings compared to the NHE.

Limitations

Despite the strengths, this study has several limitations that should be acknowledged. First, the small sample size of 10 participants may limit the generalizability of the findings. Future studies with larger sample sizes are needed to confirm these results. Second, the study included only male participants, which restricts the applicability of the findings to female athletes. Future research should include both genders to provide a more comprehensive understanding of hamstring activation across different populations. Additionally, the potential for electrode placement variability and skin impedance differences could have influenced the EMG recordings. Standardizing electrode placement and considering these factors in future studies could help mitigate these issues. Lastly, the study did not account for the potential influence of fatigue, which may have affected muscle activation during the exercises. Implementing measures to control for fatigue in future research would be beneficial.

## Conclusions

The current results revealed a significant difference in overall hamstring activation between exercises, with significantly higher muscle activation during sprinting compared to strengthening exercises. NHE showed higher activity than concentric exercise on the isokinetic machine. Although the specific activation of each muscle chief was not significant, a trend toward increased activity for the ST chief during sprinting and NHE, as well as for the BF chief during isokinetic exercise and NHE, was observed.

In conclusion, it is essential to incorporate sprinting into rehabilitation without overloading the patient, as it is a high-intensity exercise enabling maximum recruitment of the hamstrings, fundamental in injury prevention and rehabilitation. Combining sprinting with exercises such as the NHE and deadlift is essential. Despite the proven effectiveness of many exercises in preventing hamstring injuries, a notable increase in cases has been observed. This raises the question of the effective use of these exercises in rehabilitation and prevention by physiotherapists and in sports training.

## References

[1] Yeung SS, Suen AM, Yeung EW (2009). A prospective cohort study of hamstring injuries in competitive sprinters: preseason muscle imbalance as a possible risk factor. Br J Sports Med.

[2] Ekstrand J, Bengtsson H, Waldén M, Davison M, Khan KM, Hägglund M (2022). Hamstring injury rates have increased during recent seasons and now constitute 24% of all injuries in men's professional football: the UEFA Elite Club Injury Study from 2001/02 to 2021/22. Br J Sports Med.

[3] Tsaklis P, Malliaropoulos N, Mendiguchia J, Korakakis V, Tsapralis K, Pyne D, Malliaras P (2015). Muscle and intensity based hamstring exercise classification in elite female track and field athletes: implications for exercise selection during rehabilitation. Open Access J Sports Med.

[4] Howard RM, Conway R, Harrison AJ (2018). Muscle activity in sprinting: a review. Sports Biomech.

[5] Higashihara A, Nagano Y, Ono T, Fukubayashi T (2016). Relationship between the peak time of hamstring stretch and activation during sprinting. Eur J Sport Sci.

[6] Bourne MN, Williams MD, Opar DA, Al Najjar A, Kerr GK, Shield AJ (2017). Impact of exercise selection on hamstring muscle activation. Br J Sports Med.

[7] Higashihara A, Ono T, Tokutake G, Kuramochi R, Kunita Y, Nagano Y, Hirose N (2019). Hamstring muscles' function deficit during overground sprinting in track and field athletes with a history of strain injury. J Sports Sci.

[8] Iga J, Fruer CS, Deighan M, Croix MD, James DV (2012). 'Nordic' hamstrings exercise - engagement characteristics and training responses. Int J Sports Med.

[9] Sanfilippo JL, Silder A, Sherry MA, Tuite MJ, Heiderscheit BC (2013). Hamstring strength and morphology progression after return to sport from injury. Med Sci Sports Exerc.

[10] de Carvalho Froufe Andrade AC, Caserotti P, de Carvalho CM, de Azevedo Abade EA, da Eira Sampaio AJ (2013). Reliability of concentric, eccentric and isometric knee extension and flexion when using the REV9000 isokinetic dynamometer. J Hum Kinet.

[11] Cvjetkovic DD, Bijeljac S, Palija S, Talic G, Radulovic TN, Kosanovic MG, Manojlovic S (2015). Isokinetic testing in evaluation rehabilitation outcome after ACL reconstruction. Med Arch.

[12] Croisier JL (2004). Factors associated with recurrent hamstring injuries. Sports Med.

[13] Green B, Bourne MN, van Dyk N, Pizzari T (2020). Recalibrating the risk of hamstring strain injury (HSI): a 2020 systematic review and meta-analysis of risk factors for index and recurrent hamstring strain injury in sport. Br J Sports Med.

[14] Briggs KK, Lysholm J, Tegner Y, Rodkey WG, Kocher MS, Steadman JR (2009). The reliability, validity, and responsiveness of the Lysholm score and Tegner activity scale for anterior cruciate ligament injuries of the knee: 25 years later. Am J Sports Med.

[15] Hermens HJ, Freriks B, Disselhorst-Klug C, Rau G (2000). Development of recommendations for SEMG sensors and sensor placement procedures. J Electromyogr Kinesiol.

[16] Materia SI (2024). BTS Bioengineering. FREEEMG wireless surface EMG. https://www.btsbioengineering.com/products/freeemg/.

[17] Prince C, Morin JB, Mendiguchia J, Lahti J, Guex K, Edouard P, Samozino P (2020). Sprint specificity of isolated hamstring-strengthening exercises in terms of muscle activity and force production. Front Sports Act Living.

[18] Freeman BW, Young WB, Talpey SW, Smyth AM, Pane CL, Carlon TA (2019). The effects of sprint training and the Nordic hamstring exercise on eccentric hamstring strength and sprint performance in adolescent athletes. J Sports Med Phys Fitness.

[19] Ditroilo M, De Vito G, Delahunt E (2013). Kinematic and electromyographic analysis of the Nordic hamstring exercise. J Electromyogr Kinesiol.

